# Human Monoclonal scFvs that Neutralize Fribrinogenolytic Activity of Kaouthiagin, a Zinc-Metalloproteinase in Cobra (*Naja kaouthia*) Venom

**DOI:** 10.3390/toxins10120509

**Published:** 2018-12-03

**Authors:** Jirawat Khanongnoi, Siratcha Phanthong, Onrapak Reamtong, Anchalee Tungtronchitr, Wanpen Chaicumpa, Nitat Sookrung

**Affiliations:** 1 Graduate Program in Immunology, Department of Immunology, Faculty of Medicine Siriraj Hospital, Mahidol University, Bangkok 10700, Thailand; jirawatkhanongnoi@gmail.com (J.K.); peppermintpillow@hotmail.com (S.P.); 2 Center of Research Excellence on Therapeutic Proteins and Antibody Engineering, Department of Parasitology, Faculty of Medicine Siriraj Hospital, Mahidol University, Bangkok 10700, Thailand; anchalee.tun@mahidol.ac.th (A.T.); wanpen.cha@mahidol.ac.th (W.C.); 3 Department of Molecular Tropical Medicine and Genetics, Faculty of Tropical Medicine, Mahidol University, Bangkok 10400, Thailand; onrapak.rea@mahidol.ac.th; 4 Biomedical Research Incubation Unit, Department of Research, Faculty of Medicine Siriraj Hospital, Mahidol University, Bangkok 10700, Thailand

**Keywords:** cobra, human single-chain antibody variable fragments (HuscFvs), kaouthiagin, *Naja kaouthia*, snake venom metalloproteinase (SVMP), von Willebrand factor (vWF)

## Abstract

Snake venom-metalloproteinases (SVMPs) are the primary factors that disturb hemostasis and cause hemorrhage in the venomous snake bitten subjects. Kaouthiagin is a unique SVMP that binds and cleaves von Willebrand factor (vWF) at a specific peptide bond leading to inhibition of platelet aggregation, which enhances the hemorrhage. Kaouthiagin is a low abundant venom component of Thai cobra (*Naja kaouthia*); thus, most horse-derived antivenins used for cobra bite treatment do not contain adequate anti-kaouthiagin. This study aimed to produce human single-chain antibody variable fragments (HuscFvs) that bind to and interfere with kaouthiagin activity for further clinical use. Kaouthiagin was purified from *N. kaouthia*-holovenom by a single-step gel-filtration chromatography. The purified venom component was used in phage-biopanning to select the kaouthiagin-bound HuscFv-displayed-phage clones from a HuscFv-phage display library. The selected phages were used to infect Escherichia coli bacteria. Soluble HuscFvs expressed by three phage-transformed-*E. coli* clones interfered with cobra kaouthiagin binding to human vWF. Computerized simulation indicated that HuscFv of two phage-transformed *E. coli* clones formed contact interface with kaouthiagin residues at or near catalytic site and effectively inhibited fibrinogenolytic activity of the kaouthiagin. The HuscFvs have therapeutic potential as an adjunct of antivenins in treatment of bleeding caused by venomous snakebites.

## 1. Introduction

Recent estimation on worldwide incidence of venomous snakebites was 5.4 million human cases annually, mostly in Asia, Africa, and Latin America; among them 1.8–2.7 million developed clinical manifestations and 81,000 to 138,000 were fatal [1]. In Thailand in 2015, the snakebite incidence was 7.06 per 100,000 population and cobra-bites contribute the most frequent hospitalized cases [2]. *Naja kaouthia* (Thai cobra) is the most common venomous snake found in Thailand [3]. The *N. kaouthia* venom is a complex mixture of pharmacologically active molecules, comprise mainly several proteins and polypeptide components [4] including various neurotoxins [4,5], phospholipase A2 [4,6,7], cobra venom factor [4,8], cardiotoxins [4,9], cytotoxin [4], mocarhagin [4], muscarinic toxin-like protein [4], and snake venom metalloproteinases (SVMPs) [4,10].

A significant fraction of the snake-bite survivors may have long-term disabilities and poor quality of life [11]. The hemorrhage is promoted by SVMPs which are the venom enzymes responsible for local and systemic disturbance of the hemostatic system [12,13]. The SVMPs are zinc-dependent proteases, which belong to the Metzincin protein superfamily with a characteristic zinc-binding motif (HEbxHxbGbxHD) in the catalytic domain (M domain), followed by a Met-turn region (a structure contains a conserved Met residue that forms a hydrophobic basement for the three zinc-binding histidines in the consensus sequence) [14,15]. The SVMPs are classified into P-I to P-III, according to differences in their molecular sizes and domain organization [16]. The P-I class is small SVMPs; each molecule composed of a single metalloproteinase (M) domain. The P-II molecule contains two domains including M and disintegrin (D) domains; the P-III class is the high molecular mass SVMP, which composed of M domain, followed by D domain, and cysteine-rich (C) domain. P-II and P-III SVMPs are further divided into subclasses, i.e., a–d, based on their different post-translational modifications. The P-IIId (formerly P-IV) is the P-III that contains additional disulfide-linked snake C-type lectin-like (snaclec) domain [16,17,18,19]. The SVMPs need zinc ions for their proteolytic activity and calcium ions for structural stabilization [18,19]. They induce hemorrhage by degrading directly protein components of the endothelial cells (e.g., integrin and cadherin) and vascular basement membrane, e.g., collagen IV, laminin, nidogen, and proteoglycan perlecan [20]. They also cleave and disturb proteins involved in hemostasis or blood coagulation, e.g., fibrinogen, factor X, prothrombin, and von Willebrand factor (vWF) [18,21,22,23].

Kaouthiagin is a P-III SVMP, which contained in minute amount in the *N. kaouthia* venom [22]. The protein binds and degrades vWF at a specific peptide bond, i.e., between Pro708 and Asp709, to create vWF fragments and the vWF multimerization, resulting in loss of the vWF-mediated-platelet aggregation and collagen binding activity; thus, enhances hemorrhage [22]. Most of antivenins against cobra venom contains negligible amount of anti-kaouthiagin [4]. Thus, this study aimed to produce human single-chain antibody variable fragments (HuscFvs) that neutralize the activity of kaouthiagin for use as an adjunct of antivenins in treatment of venomous snakebites.

## 2. Results

### 2.1. Purified Cobra Kaouthiagin

**Figure 1:**
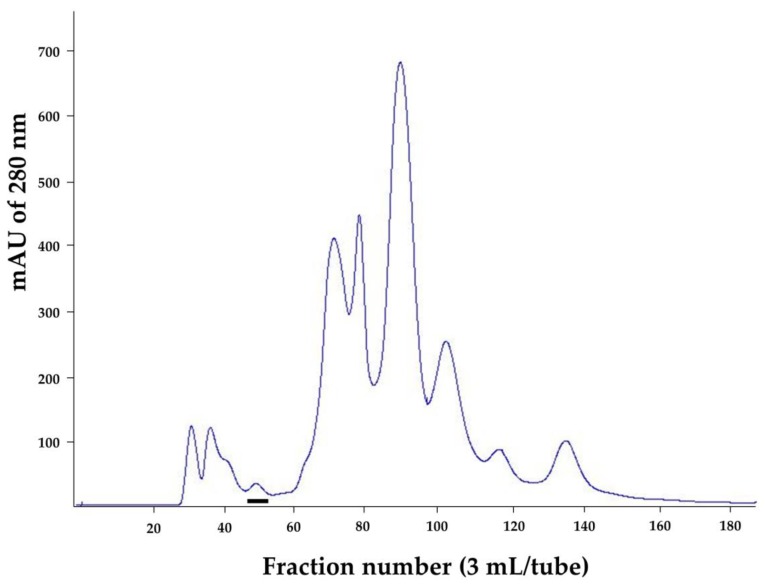
Protein profile of *N. kaouthia* holovenom separated by a Sephacryl S-200 column chromatography. *X* axis, fraction number (3 mL/tube). *Y* axis, mAU of 280 nm (milli-absorbance units at 280 nanometers). The bar indicates the fractions that showed von Willebrand (vWF)-binding activity, i.e., presumptively kaouthiagin.

**Figure 2:**
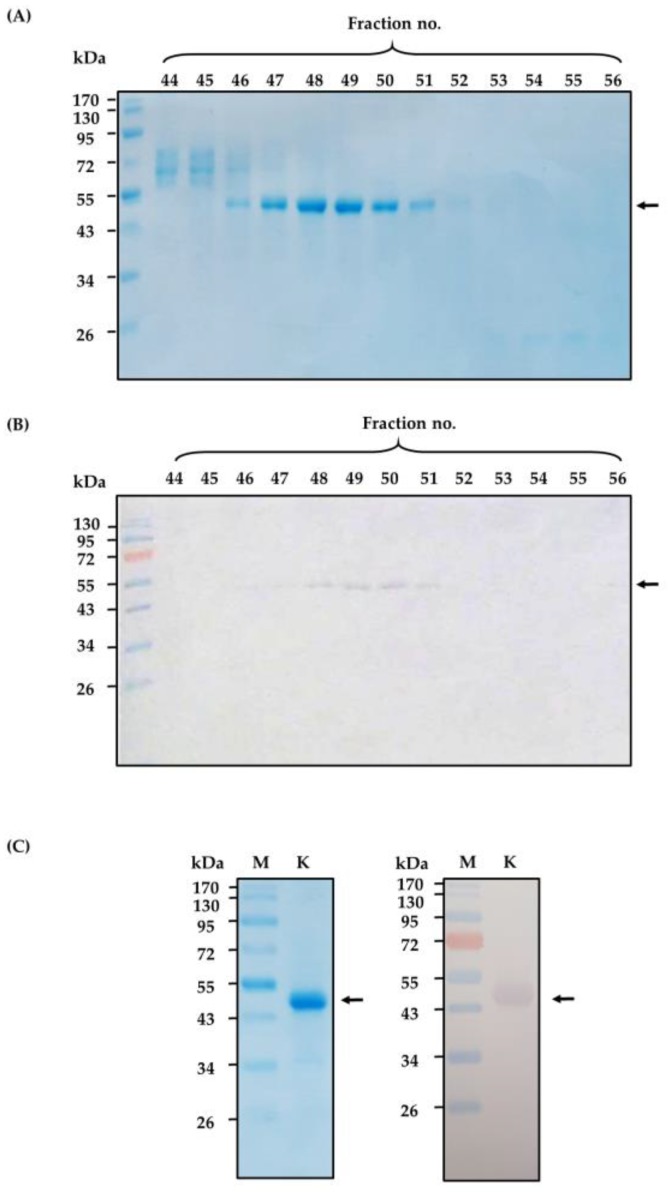
(**A**) Sephacryl S-200 column chromatographic fractions no. 44–56 were separated by 14% under non-reducing condition and the presumptive kaouthiagin bands were revealed in fractions 46–52 at ~50 kDa after Coomassie Brilliant Blue G-250 (CBB) staining. (**B**) Western blotting patterns of the SDS-PAGE-separated fractions 44–56 probed with 1 μg/mL of recombinant human von Willebrand factor (vWF); the vWF bound to the separated venom components in fractions 46–52, indicating that vWF-bound protein is *N. kaouthia* kaouthiagin which was subsequently verified by LC-MS/MS and orthologous protein database search. (**C**) SDS-PAGE-separated-concentrated kaouthiagin after CBB staining (left panel) and Western blot pattern of the concentrated kaouthiagin probed with vWF (right panel). K, purified kaouthiagin with an apparent molecular weight of ~50 kDa (arrow). M of all panels, standard molecular weight proteins.

*N. kaouthia* holovenom was fractionated (3 mL/fraction) through the Sephacryl S-200 chromatography and the chromatographic profile of the venom proteins is shown in Figure 1. The eluted fractions that contained proteins (as determined by spectrometry at OD_280nm_) were subjected to SDS-PAGE and protein staining. Fractions 46–52 were found to contain protein bands of ~50 kDa in SDS-PAGE, which is the molecular size of the venom kaouthiagin (Figure 2A). These protein bands were bound by 6× His-tagged-recombinant human von Willebrand factor (6×-His-r-hvWF) (Figure 2B). The r-hvWF-binding fractions were pooled and concentrated (Figure 2C). The LC-MS/MS verified that the preparation was cobra kaouthiagin (Table S1).

### 2.2. Soluble HuscFvs That Bound to Kaouthiagin

**Figure 3:**
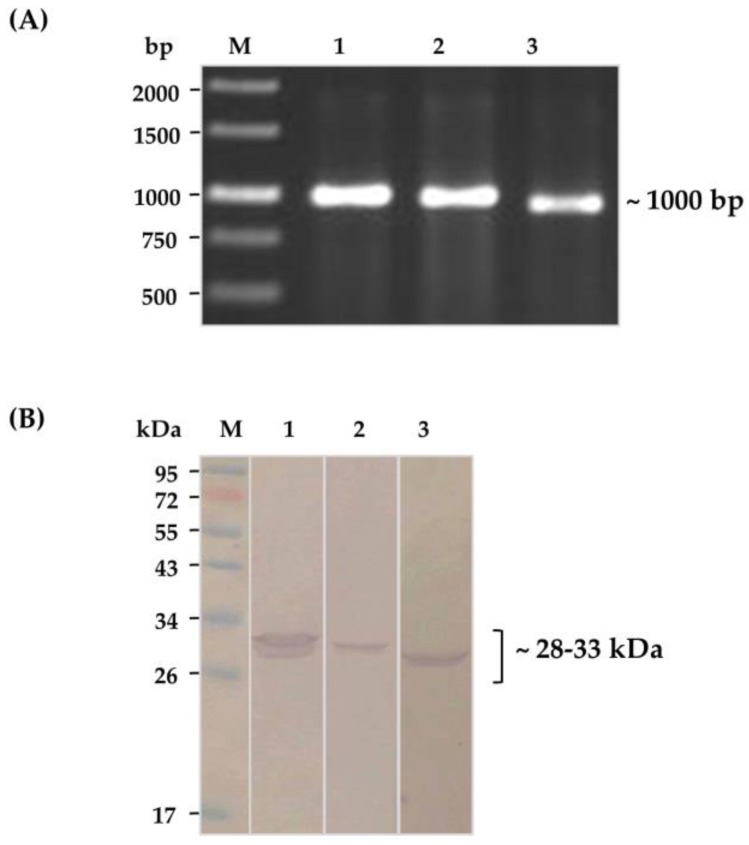
(**A**) Representative DNA amplicons of genes coding for HuscFvs (*huscfvs*) from phage infected *E. coli* clones 1, 2 and 3 at ~1000 bp. M, Standard DNA ladder. Numbers at the left are DNA sizes in base pairs (bp). (**B**) HuscFvs (~28–33 kDa) expressed from the *E. coli* clones 1, 2, and 3, respectively. M, molecular weight protein standard. Numbers at the left are proteins sizes in kDa.

Purified kaouthiagin was used as bait in phage bio-panning to fish-out HuscFv-displayed phage clones that bound to the protein from the HuscFv phage display library [24]. The kaouthiagin-bound phages were used to infect HB2151 *E. coli.* Ninety-one phage-infected-*E. coli* colonies that grew on the selective agar plate were screened for the presence of *huscfvs* (~1000 bp) by direct colony PCR, and 44 colonies were positive; their representatives are shown in Figure 3A. Sixteen of these *E. coli* clones could express soluble HuscFvs (~28–33 kDa); representatives are shown in Figure 3B.

### 2.3. Binding of the Soluble HuscFvs to Kaouthiagin

**Figure 4:**
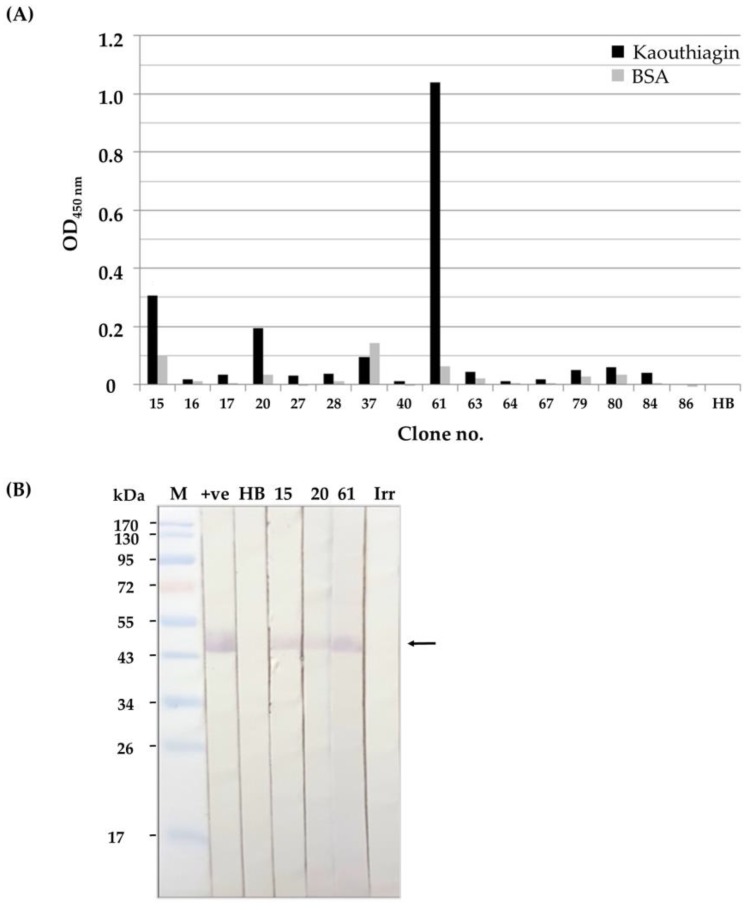
(**A**) Indirect enzyme-linked immunosorbent assay (ELISA) for determining the binding of HuscFvs expressed from various phage-infected *E. coli* clones to purified kaouthiagin. HuscFvs in lysates of *E. coli* clones no. 15, 20, and 61 gave significant ELISA signals (OD_405nm_) to kaouthiagin more than two times higher than bovine serum albumin (BSA) (antigen control). *E. coli* HB2151 (HB), lysate of normal *E. coli* HB2151 (background binding control). (**B**) Western blot patterns of kaouthiagin (~50 kDa) probed with lysates of *E. coli* clones 15, 20, and 61 which contained HuscFvs. The HuscFvs bound to the kaouthiagin and appear as bands at ~50 kDa. M, standard molecular weight proteins; +ve, kaouthiagin blotted strip probed with vWF (positive binding control); HB, kaouthiagin blotted strip probed with lysate of normal HB2151 *E. coli* (background control); Irr, kaouthiagin blotted strip probed with lysate of HB2151 *E. coli* containing irrelevant HuscFv (negative binding control).

Soluble HuscFvs in lysates of the 16 *huscfv*-phagemid-transformed HB2151 *E. coli* clones were tested for kaouthiagin binding by indirect enzyme-linked immunosorbent assay (ELISA) and Western blot analysis. By the indirect ELISA, lysates of three clones (nos. 15, 20, and 61) bound to kaouthiagin and gave the significant ELISA signal above the binding to bovine serum albumin (BSA), which was used as antigen control after subtracting with the background binding to lysate of original HB2151 *E. coli* (HB) (Figure 4A). The HuscFvs of these clones also bound to SDS-PAGE-separated-kaouthiagin band (~50 kDa) on the blotted strips of nitrocellulose membrane (NC) (Figure 4B).

### 2.4. HuscFvs-Mediated Inhibition of Binding of Kaouthiagin to vWF

**Figure 5:**
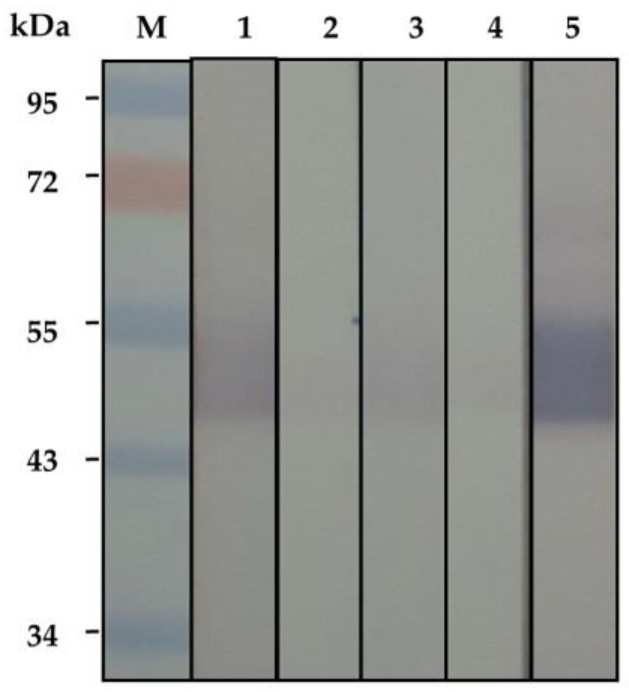
Results of HuscFv-mediated inhibition of von Willebrand factor (vWF) binding to kaouthiagin by Western blot analysis. Lane M, Pre-stained standard protein marker; lane 1, SDS-PAGE-separated kaouthiagin blotted strip probed with lysate of original HB2151 *E. coli* before adding r-hvWF (background inhibition control); lanes 2–4, SDS-PAGE-separated kaouthiagin blotted strip probed with lysates of HB2151 *E. coli* clones no. 15, 20 and 61, respectively, before incubating with r-hvWF; lane 5, SDS-PAGE-separated kaouthiagin blotted strip directly probed with recombinant human von Willebrand factor (r-hvWF) (non-inhibition control). Numbers at the left are protein masses in kDa. Pre-incubating the SDS-PAGE-separated kaouthiagin blotted NC strips with HuscFvs of *E. coli* clones 15, 20 and 61 before probing with r-hvWF was found to inhibit the r-hvWF binding to the kaouthiagin, i.e., faint bands in lanes 2–4 compared to lanes 1 and 5.

The kaouthiagin-bound HuscFvs were checked for their ability to inhibit binding of r-hvWF to kaouthiagin by Western blotting. In the inhibition test of Figure 5, pre-incubating of the SDS-PAGE-separated kaouthiagin blotted NC strips with HuscFvs from *E. coli* clones 15, 20 and 61 inhibited binding of the r-hvWF to the kaouthiagin, i.e., the r-hvWF-kaouthiagin bands at ~50 kDa were fainter compared to the respective bands without HuscFvs.

### 2.5. HuscFv-Mediated Inhibition of Fibrinogenolytic Activity of Kaouthiagin

**Figure 6:**
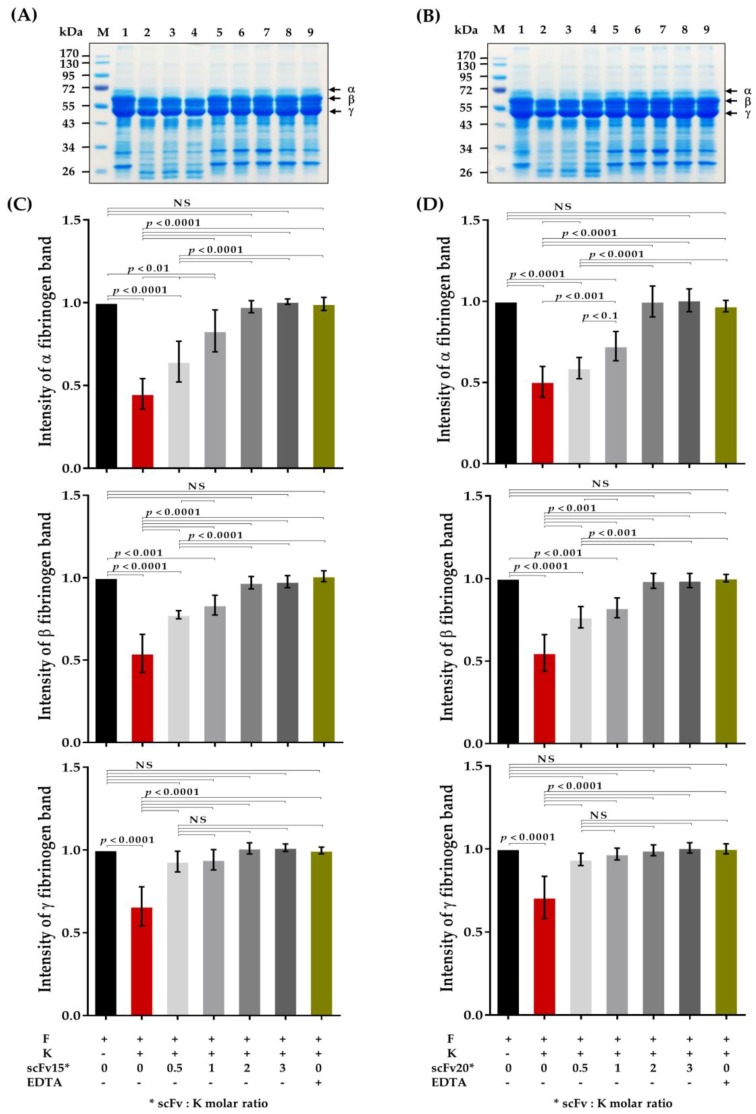
Inhibition of fibrinogenolytic activity of kaouthiagin by (**A**) HuscFv clone 15 and (**B**) HuscFv clone 20. Kaouthiagin (K) was pre-incubated with HuscFv of individual E. coli clones (scFv15/scFv20) at various molar ratios before bovine fibrinogen (F) was added to individual K-HuscFv mixtures. After incubation at 37 °C for 6 h, the samples were analyzed by SDS-PAGE and intensities of bands of α-, β- and γ- fibrinogens were determined spectrometrically. Lane M, pre-stained protein ladder; lane 1, F alone; lanes 2, F + K (digestion control or negative inhibition control); lanes 3–6, F added with mixtures of K-scFv at molar ratios of 0.5, 1, 2, and 3, respectively; lane 7, F incubated with scFv; lane 8, F added with mixture of K and EDTA (positive inhibition control); and lane 9, F mixed with EDTA. (**C**) and (**D**) are band densities of α-fibrinogen (α; upper panels), β-fibrinogen (β; middle panels) and γ-fibrinogen (γ; lower panels) fibrinogens from individual treatments of (**A**) and (**B**), respectively, as determined by spectrophotometer. The horizontal lines above the bar graphs indicate *p*-values; *p* < 0.05 indicates significant difference of band intensities of different treatments. At K:scFv molar ratios 2 and 3, the HuscFv15 and HuscFv20 completely inhibited fibrinogenolytic activity of the kaouthiagin.

Results of the experiments to demonstrate ability of the HuscFvs in inhibiting fibrinogenolytic activity of kaouthiagin are shown in Figure 6. The *N. kaouthia* kaouthiagin cleaved all forms (α, β, and γ) of fibrinogen (F) (Figure 6). The fibrinogenolytic activity of the kaouthiagin was inhibited by the HuscFvs in a dose-dependent manner after pre-incubating with HuscFv15 (Figure 6A) and HuscFv20 (Figure 6B) at the kaouthiagin:HuscFv molar ratios 0.5, 1, 2 and 3 before the mixtures were added to the fibrinogen.

### 2.6. Residues and Regions of the Kaouthiagin That Were Bound by the HuscFvs

**Figure 7:**
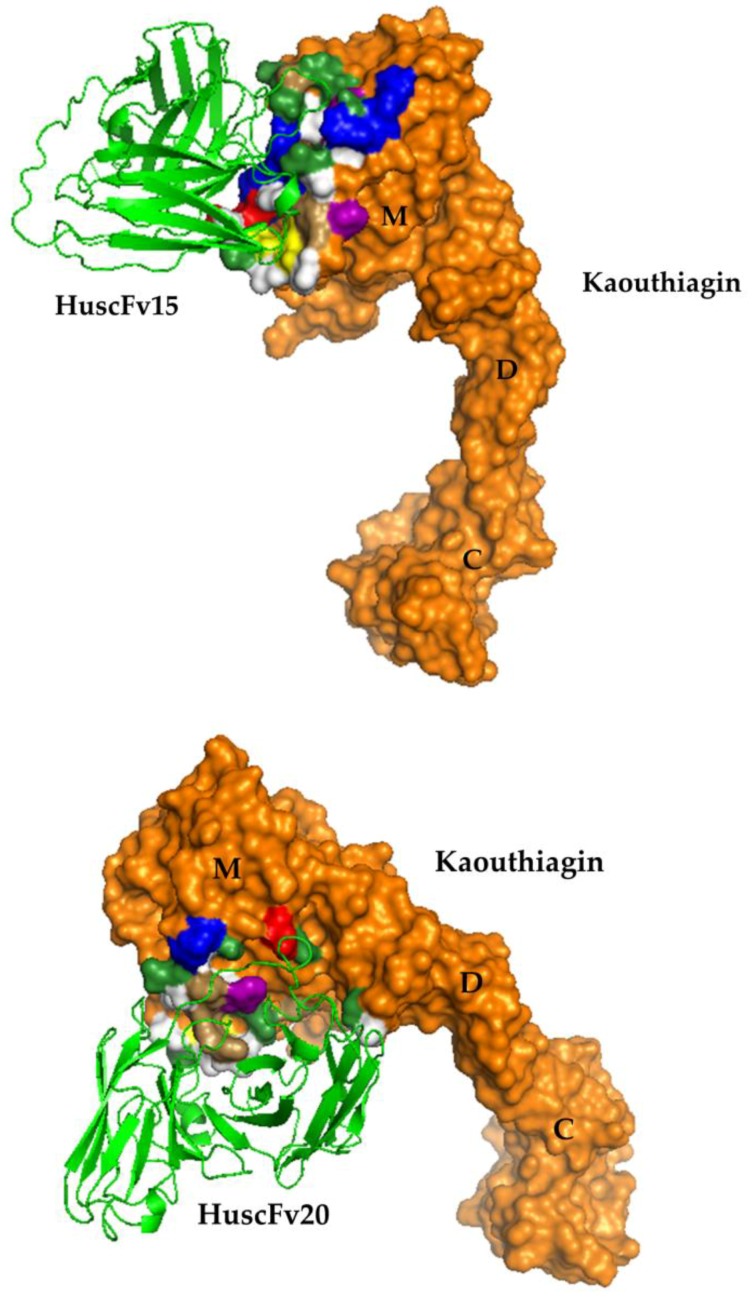
Computerized simulation to reveal regions on the kaouthiagin bound by HuscFvs. Both HuscFv15 and HuscFv20 formed contact interface with several residues near catalytic site of the M domain. Details of the interaction are given in Table 1. M, metalloproteinase domain, D, disintegrin-like domain, C, cysteine-rich domain. The kaouthiagin amino acids are colored according to CINEMA color scheme: polar negative E is red; polar positive H, K, and R are blue; polar neutral S, T, N, and Q are green; non-polar aromatic F and Y are purple/magenta; non-polar aliphatic A, V, L, and I are white, P is brown, and C is yellow.

The deduced amino acid sequences of the *huscfvs* of clones 15, 20, and 61 were predicted for immunoglobulin framework regions (FRs) and complementarity determining regions (CDRs) by International ImMunoGeneTics (IMGT) tool website (www.imgt.org). The deduced HuscFv sequences of clones 15 and 20 were complete sequences of single-chain antibodies, i.e., each sequence contained 4 FRs and 3 CDRs of variable heavy chain domain (VH) and variable light chain domain (VL) which were linked by (G_4_S)_3_. The sequences showed high identity with human VH and VL sequences of the database (Supplementary Table S2). The deduced HuscFv sequences of clone 61 was not complete, i.e., lacks VL-CDR1, VL-CDR2 and VL-FR2 and some amino acids in VL-FR1 and VL-FR3 (data not shown) and low percentage of amino acid homology to human immunoglobulin (Supplementary Table S2). Thus, the HuscFv61 was not tested further. The presumptive residues and regions of kaouthiagin that were bound by the HuscFv15 and HuscFv 20 are shown in Figure 7, and Table 1. HuscFvs of both clones formed contact interface with kaouthiagin residues at the specific surface region of SVMPs that locates near to the M domain catalytic site.

## 3. Discussion

Most antivenins against Elapidae venoms usually contained predominantly neutralizing antibodies to lethal/major venom toxins and little amount of antibodies to low abundant venom components although these components contribute also to serious adverse pharmacological effects in the bitten subjects. SVMPs are abundant in Viperidae family of venomous snakes, but they are present only in minute amounts in venoms of the Elapidae family, such as, *Naja kaouthia* and *Ophiophagus hannah* (king cobra) [4,25]. Recently, *N. kaouthia* venom was found to contain a unique vWF-binding protein, named kaouthiagin, that binds and cleaves vWF at a specific peptide bond resulting in a loss of the platelet- and collagen- binding activity of the vWF; hence enhance hemorrhage at the snake biting site [22]. Kaouthiagin can also cause damage of the basement membrane due to cytotoxicity to the endothelial cells [12,26]. Currently, several substances have been used for inhibition of hemorrhage caused by SVMPs [27]. These include animal derived antivenins, natural venom inhibitors from animal sera and some plants, as well as electric current treatment [27,28]. In this study, recombinant human single-chain antibody variable fragments (HuscFvs) that bound to kaouthiagin and inhibited vWF binding and fibrinogenolytic activities of the toxin were produced for use as a supplement of the existing antivenins that contain inadequate anti-hemorrhagic antibodies. The same strategy reported in this communication can be applied also for in vitro generation of antibodies to other low amount, low immunogenic, highly toxic venom components.

*N. kaouthia* kaouthiagin with more than 90% purity was successfully purified from the *N. kaouthia* holovenom by using a highly convenient one-step size exclusion chromatography. Fully human single-chain antibodies that bound to the kaouthiagin were prepared by using phage display technology. HuscFvs of two phage transformed-*E. coli* clones (15 and 20) that showed complete sequence of single-chain antibody fragments, i.e., FRs 1-4 and CDR1-3 of both VH and VL domains linked together by (G_4_S)_3_ inhibited kaouthiagin-von Willebrand factor interaction as well as interfered with the kaouthiagin fibrinogenolytic activity. Because the HuscFvs were products of human immunoglobulin genes, they should not have immunogenicity in the human recipients (without any untoward reactions such as anaphylaxis or serum sickness that usually occur when animal-derived antibodies were used in snakebite treatment). The antibody fragments are five-times smaller than the conventional IgG; thus, they should have high penetrating activity into the surrounding tissue when injected locally at the biting site and thus can rapidly exert the therapeutic effect. Computerized simulation indicated that the HuscFv15 formed contact interface with the kaouthiagin residues H159, A 162, C166, P170, L174, and K176 while HuScFv20 bound to kaouthiagin residues A162, S163, C166, I167, P168, C169, and P170. The residues 153–176 of SVMPs have been known to be a specific surface region that determined whether the SVMPs possess strong or weak hemorrhagic activity as the region is related to ability to bind appropriate substrate and also critical for protein-protein interface [15,29,30]. This loop region lies close to the M domain catalytic site [15]. Therefore, binding of the HuscFv15 and HuscFv20 to this part of the kaouthiagin molecule should explain their ability in inhibiting the fibrinogenolytic activity of the venom component. The HuscFv15 also bound to H159 which is one of the residues of the catalytic site. Although the modelling results are speculative and need to be confirmed by experimental data, the results of this study suggest that the HuscFvs deserve further development for adjunctive therapy of venomous snakebite.

## 4. Materials and Methods

### 4.1. N. kaouthia Holovenom and Kaouthiagin Purification

*N. kaouthia* holovenom in lyophilized form was purchased from Queen Saovabha Memorial Institute (QSMI), Thai Red Cross, Bangkok, Thailand. The lyophilized venom (300 mg) was dissolved in 5 mL of 0.05 M ammonium bicarbonate buffer, pH 7.8. The solution was centrifuged at 12,000× *g*, 4 °C for 10 min. The soluble part of venom was filtered through sterile 0.45 µm before applying to a Hiprep 26/60 Sephacryl S-200 chromatography column (Sephacryl^®^ S-200 HR, GE Healthcare, Uppsala, Sweden). Elution (3 mL/fraction) was carried out with the buffer (50 mM of ammonium bicarbonate buffer, pH 7.8) at a flow rate of 0.3 mL/min. Protein concentrations of all eluted fractions were measured and analyzed by SDS-PAGE and Coomassie Brilliant Blue G-250 (CBB) staining. The presence of kaouthiagin in individual fractions were determined by probing the SDS-PAGE-separated fractions with 6× His-tagged-recombinant human vWF (r-hvWF) (VWF-894H, Creative Biomart, Ramsey road, Shirley, NY, USA), followed by mouse anti-6× His antibody, goat-anti-mouse IgG-alkaline phosphatase (AP) conjugate (Southern Biotech, Birmingham, AL, USA) and AP substrate (BCIP/NBT; KPL, Seracare Life Sciences, Milford, MA, USA), respectively, with washing and incubation between the steps. The r-hvWF-bound fractions were collected, dialyzed against distilled water, concentrated and subjected to LC-MS/MS for kaouthiagin verification.

### 4.2. Production of Kaouthiagin-Bound HuscFvs

The HuscFv phage display library was constructed previously [24] using total RNA extracted from peripheral blood mononuclear cells of 60 healthy, non-immunized Thai blood donors and messenger RNA (mRNA) was reverse transcribed to cDNA. The gene fragments encoding VH and VL domains of immunoglobulins were PCR amplified using the cDNA as template and 14 forward and 3 reverse degenerate primers designed from all families of human immunoglobulin genes [24]. The cDNA amplicons were ligated into a pCANTAB5E phagemid vector and introduced into competent TG1 *E. coli* cells. The complete phage particles displaying human scFvs (HuscFvs) with integrated *huscfvs* in the phage genomes were rescued from by co-infecting the *huscfv*-phagemid transformed *E. coli* with a helper phage.

One microgram of the purified kaouthiagin was coated onto an ELISA well surface. The HuscFv phage display library was added to incubate with the immobilized antigen. After removing the antigen-unbound phages by washing with 0.05% Tween-20 in PBS (PBST), the antigen-bound phages were used to infect HB2151 *E. coli.* The phage transformed *E. coli* bacteria were grown on a selective 2× YT (yeast extract tryptone) agar containing ampicillin and glucose (2× YT-AG). The pCANTAB-5E phagemid-transformed *E. coli* colonies were screened for the presence of the HuscFv coding genes (*huscfvs*) by direct colony PCR using R1 and R2 phagemid-specific primers [24]. The *E. coli* harboring *huscfvs* which provided expected DNA amplicon of ~1000 bp were grown under isopropyl β-D-1-thiogalactopyranoside (IPTG) induction condition and their lysates were screened for soluble E-tagged-HuscFvs by Western blot analysis using anti-E-tag (Abcam, Cambridge, UK) as the antibody detection reagent.

### 4.3. Binding of the HuscFvs to Kaouthiagin

The HuscFvs in lysates of huscfv-phagemid transformed *E. coli* were tested for binding to kaouthiagin by indirect ELISA and Western blotting. For ELISA, wells were coated with 1 µg of purified kaouthiagin; bovine serum albumin (BSA) served as antigen control. The antigen-coated wells were incubated individually with HuscFv-containing *E. coli* lysates. Lysate of original HB2151 *E. coli* (HB) was used as background (negative HuscFv) binding control. The antigen-bound HuscFvs were detected by using rabbit anti-E-tagTM monoclonal antibody (Abcam, Cambridge, UK), goat anti-rabbit IgG-horseradish peroxidase (HRP) conjugate (Southern Biotech, Bermingham, AL, USA) and ABTS substrate (KPL, Seracare Life Sciences, Milford, MA, USA), respectively, with incubation and washing between the steps.

For Western blot analysis, kaouthiagin was subjected to 14% SDS-PAGE and then blotted onto nitrocellulose membrane (NC). After blocking with 5% skimmed milk in 0.05% Tween-20 in TBS (TBST), the NC was stripped vertically and blotted strips were incubated with lysates of individual HB2151 *E. coli* clones containing HuscFvs. After washing with TBST, the strips were probed with rabbit anti-E tag antibody, goat-anti-rabbit IgG-AP conjugate (Southern Biotech) and BCIP/NBT (KPL) substrate, respectively, with incubation and washing between the steps. Negative and positive binding controls were kaouthiagin blotted strips incubated with normal HB2151 *E. coli* lysate (HB) and r-hvWF, respectively.

### 4.4. HuscFv-Mediated Inhibition of Kaouthiagin Binding to Human vWF (Binding Inhibition Assay)

NC strips blotted with SDS-PAGE-separated kaouthiagin were blocked with 5% skimmed milk before incubating with lysates of individual *E. coli* clones containing soluble HuscFvs. Lysate of normal HB2151 was used as negative inhibition control. After washing with TBST, each strip was immersed in a solution of 1 µg/mL of 6× His-r-hvWF. Mouse anti-6× His antibody (Abcam, Cambridge, MA, USA), goat anti-mouse IgG-AP-conjugate (Southern Biotech) and BCIP/NBT substrate (KPL) were used for detecting the r-hvWF on the strips. Non-inhibition control, i.e., direct binding of r-hvWF to the kaouthiagin, was included in the assay.

### 4.5. Inhibition of Fribrinogenolytic Activity of Kaouthiagin by HuscFvs

Fibrinogenolytic activity of the kaouthiagin was evaluated using the method described previously [31] with modification. Briefly, 50 µg of the bovine fibrinogen (Sigma-Aldrich, St. Louis, MO, USA) were mixed with 2 µg of kaouthiagin in 40 μL of 10 mM Tris-HCl buffer (pH 7.4) containing 10 mM NaCl and 5 mM CaCl_2_ and the mixture was incubated at 37 °C for 2 h. The bovine fibrinogen in buffer alone served as negative fibrinogenolytic control. The enzymatic reaction was terminated by adding denaturing buffer containing 0.5 M Tris-HCl (pH 8.8), 20% glycerol, 10% sodium dodecyl sulfate, 0.05% bromophenol blue and 10 mM β-mercaptoethanol, followed by boiling at 100 °C for 10 min. The samples were analyzed by 12% SDS-PAGE and CBB staining.

For the HuscFv-mediated inhibition of the kaouthiagin fibrinogenolytic activity, the kaouthiagin (2 µg) was pre-incubated with HuscFvs of individual *E. coli* clones at different kaouthiagin:HuscFv molar ratios, i.e., 0.5, 1, 2 and 3, at 37 °C for 1 h before the mixtures were subjected to the fibrinogenolytic assay as above. EDTA (10 mM) was used as positive kaouthiagin-inhibition control. Bovine fibrinogen mixed with kaouthiagin was negative inhibition control and fibrinogen in buffer alone was non-fibrinogenolytic control. Three independent experiments were performed.

### 4.6. Computerized Simulation to Predict the Kaouthiagin Regions and Residues Bound by the HuscFvs

The nucleotide sequences of the kaouthiagin-bound HuscFvs were determined by DNA sequencing. The 2D graphical representations of the HuscFvs and their complementarity determining regions (CDRs) and the immunoglobulin framework regions (FRs) were predicted by using the IMGT software in www.imgt.org. The deduced amino acids sequences of HuscFvs were subjected to Iterative Threading ASSEmbly Refinement (I-TASSER) online service for protein modeling. The available kaouthiagin crystal structure (PDB ID: 3K7N) was used as a specific template to guide the I-TASSER for modeling of the 3D structure of kaouthiagin from the deduced amino acid sequence. The modeled protein structures were subsequently refined by ModRefiner (version 2.3.0, Center for Computational Medicine and Bioinformatics, University of Michigan, Ann Arbor, MI, USA; http://zhanglab.ccmb.med.umich.edu/ModRefiner/) and Fragment-Guided Molecular Dynamics (FG-MD, Center for Computational Medicine and Bioinformatics, University of Michigan, Ann Arbor, MI, USA; http://zhanglab.ccmb.med.umich.edu/FG-MD/), respectively, to make the initial models closer to their native structures and improved the local geometry of the structures by removing the steric clashes. ClusPro 2.0 Server (ABC Group and Structural Bioinformatics Lab, Boston University, Boston, MA and Stony Brook University, NY, USA) was used to predict the interaction between the kaouthiagin and HuscFvs [32]. The largest cluster size with minimal local energy and a near-native state of the protein conformations was chosen for each docking. The visualize protein structure models and the molecular interactions were generated by the Pymol software of The PyMOL Molecular Graphics System (Version 1.3r1 edu, Schrodinger, LLC, New York, NY, USA).

### 4.7. Statistical Analysis

Means and standard deviations of three independent experiments were used for comparison between tests and controls. *p* < 0.05 of the unpaired *t*-test was considered significantly different.

## Figures and Tables

**Table 1:** Presumptive residues of kaouthiagin M domain that formed contact interface with HuscFv15 and HuscFv20.

Kaouthiagin Residue	Amino Acid(s)	Domain(s)	Interactive Bond(s)
**HuscFv15**			
N112	S193	VL-CDR3	Hydrogen
V116	Y101/ I103	VH-CDR3/CDR3	Hydrophobic/hydrophobic
Y136	Y101	VH-CDR3	Hydrogen, hydrophobic and aromatic-aromatic
N137	I103	VH-CDR3	Hydrogen
R139	D102	VH-CDR3	Hydrogen and ionic
L142	Y101	VH-CDR3	Hydrophobic
T146	Y101	VH-CDR3	Hydrogen
H159	S197	VL-FR3	Hydrogen
A162	I115	VH-CDR3	Hydrophobic
C166	G25	VH-CDR1	Hydrogen
P170	F26	VH-CDR1	Hydrophobic
L174	F26/Y31/I115	VH-CDR1/CDR1/CDR3	Hydrophobic/hydrophobic/hydrophobic
K176	D110/D114/Y101	VH-CDR3/CDR3	Hydrogen and ionic/hydrogen/hydrogen and cationic-π
T178	Y31/S99	VH-CDR1/CDR3	Hydrogen/hydrogen
**HuscFv20**			
E43	K64	VH-FR3	Hydrogen and ionic
A162	S107/I174	VH-CDR3/VL-CDR1	Hydrogen
S163	S107	VH-CDR3	Hydrophobic
C166	R31	VH-CDR1	Hydrogen
I167	F52/R100/W239	VH-CDR2/CDR3/VL-CDR3	Hydrophobic/hydrogen/hydrophobic
P168	F52/Y236/W239	VH-CDR2/VL-CDR3/CDR3	Hydrophobic/hydrophobic/hydrogen and hydrophobic
C169	Y236	VL-CDR3	Hydrogen
P170	W239/P240	VL-CDR3/CDR3	Hydrogen/hydrophobic
A181	W239	VL-CDR3	Hydrophobic
F182	W239	VL-CDR3	Hydrophobic and aromatic-aromatic
Q183	E56	VH-CDR2	Hydrogen

## Supplementary Materials

The following are available online at http://www.mdpi.com/2072-6651/10/12/509/s1, Table S1: LC-MS/MS Mascot result of in-gel tryptic digestion of recombinant human von Willebrand factor (r-hvWF)-binding protein of this study searching against NCBInr database, Table S2: Percent amino acid homology of the HuscFv sequences from *E. coli* clones 15 and 20 with the closest human V regionorks of the database.
