# Supplementary material for: Human Monoclonal scFvs that Neutralize Fribrinogenolytic Activity of Kaouthiagin, a Zinc-Metalloproteinase in Cobra (Naja kaouthia) Venom
Source: Toxins (Basel). 2018 Dec 3;10(12):509. doi: 10.3390/toxins10120509 (PMC6315812; doi:10.3390/toxins10120509)
Supplement: Supplementary file 1 [file toxins-10-00509-s001.pdf]

Supplementary Materials: Human Monoclonal scFvs that Neutralize Fribrogenolytic Activity of Kaouthiagin, a Zinc-Metalloproteinase in Cobra (*Naja kaouthia*) Venom

Jirawat Khanongnoi, Siratcha Phanthong, Onrapak Reamtong, Anchalee Tungtronchitr, Wanpen Chaicumpa and Nitat Sookrung

Table S1. LC-MS/MS Mascot result of in-gel tryptic digestion of recombinant human von Willebrand factor (r-hvWF)-binding protein of this study searching against NCBInr database.

Protein	Orthologous protein of database	Accession no.	Molecular mass	Protein score	Matched peptide sequence (score)
Naja kaouthia venom component that bound to r-hvWF	Hemorrhagic metalloproteinase-disintegrin-like kaouthiagin of *Naja kaouthia*	P82942	44463 Da	54	FNGAGAECR (54.34) NGHPCQNNQGYCYNGK (14.3) GCFDLNMRGDDGSFCR (15.55)

Table S2. Percent amino acid homology of the HuscFv sequences from *E. coli* clones 15 and 20 with the closest human V regionorks of the database.

HuscFv clone no.		The closest human V region	Percentage of amino acid homology with huamn FRs		
			FR1	FR2	FR3
15	VH	X92288 IGHV3-7*02	72.00	100.00	100.00
	VL	X59315 IGKV1*01	93.31	88.24	97.22
20	VH	M99648 IGH V2-26*01	96.00	100.00	100.00
	VL	M23090 IGKV3-15*01	7.69	94.12	91.67
61	VH	AC244456 IGHV3-30-3*01	44.00	94.12	78.95
	VL	X59315 IGKV1-39*01	3.85	5.88	91.67

Asterisk followed by two numbers indicates the allele polymorphism. FRs, Framework regions
